# Eye Movements as Proxy for Visual Working Memory Usage: Increased Reliance on the External World in Korsakoff Syndrome

**DOI:** 10.3390/jcm12113630

**Published:** 2023-05-23

**Authors:** Sanne Böing, Antonia F. Ten Brink, Alex J. Hoogerbrugge, Erik Oudman, Albert Postma, Tanja C. W. Nijboer, Stefan Van der Stigchel

**Affiliations:** 1 Experimental Psychology, Helmholtz Institute, Utrecht University, 3584 CS Utrecht, The Netherlands; a.f.tenbrink@uu.nl (A.F.T.B.); a.j.hoogerbrugge@uu.nl (A.J.H.);; 2 Korsakoff Center of Expertise Slingedael, 3086 EZ Rotterdam, The Netherlands; 3 Center of Excellence for Rehabilitation Medicine, University Medical Center Utrecht and De Hoogstraat Rehabilitation, 3583 TM Utrecht, The Netherlands

**Keywords:** visual working memory, external memory, acquired brain injury, copy task, eye movements, Korsakoff syndrome, cognitive offloading

## Abstract

In the assessment of visual working memory, estimating the maximum capacity is currently the gold standard. However, traditional tasks disregard that information generally remains available in the external world. Only when to-be-used information is not readily accessible, memory is taxed. Otherwise, people sample information from the environment as a form of cognitive offloading. To investigate how memory deficits impact the trade-off between sampling externally or storing internally, we compared gaze behaviour of individuals with Korsakoff amnesia (*n* = 24, age range 47–74 years) and healthy controls (*n* = 27, age range 40–81 years) on a copy task that provoked different strategies by having information freely accessible (facilitating sampling) or introducing a gaze-contingent waiting time (provoking storing). Indeed, patients sampled more often and longer, compared to controls. When sampling became time-consuming, controls reduced sampling and memorised more. Patients also showed reduced and longer sampling in this condition, suggesting an attempt at memorisation. Importantly, however, patients sampled disproportionately more often than controls, whilst accuracy dropped. This finding suggests that amnesia patients sample frequently and do not fully compensate for increased sampling costs by memorising more at once. In other words, Korsakoff amnesia resulted in a heavy reliance on the world as ‘external memory’.

## 1. Introduction

To objectify memory complaints and estimate memory functioning following acquired brain injury, traditionally, patients are asked to memorise an increasing number of briefly presented stimuli and are assessed on how many items are retained (e.g., change detection task [1]; Corsi block-tapping task [2,3]; digit span task [4]). The maximum storage capacity is then used to dissociate between normative and deviant performance, and subsequently to guide diagnosis and understand patient (dys)functioning in daily situations. However, such tests disregard that in daily life information typically remains available in the external world. We can easily sample information by making eye movements, using the environment as our ‘external memory’. Sampling information from the external world is reminiscent of cognitive offloading, where people decide to perform a physical action in order to reduce the internal cognitive effort to carry out a task [5,6,7,8]. Similarly, as making an eye movement is easy and quick, people generally tend to sample information instead of memorising it. Sampling information that is easily accessible in the external world reduces the need to use the maximum VWM storage capacity. Contrarily, when it is difficult or costly to access information in the external world, sampling rates decrease and reliance on internal VWM storage increases [9,10,11,12,13,14]. This implies a cost-efficient trade-off between sampling and storing. Consequently, the existence of such a trade-off suggests that the maximum storage capacity is often not used in natural behaviour. Capacity scores might therefore not translate to memory functioning in daily life [15]. A better way to approximate memory functioning in daily life might be by assessing sampling behaviour. The overarching aim of this study was therefore to assess whether eye movement patterns during the execution of a memory task can serve as a proxy for VWM use in individuals with and without memory impairments. To this end, we compared the gaze behaviour of individuals without memory impairments and patients with Korsakoff amnesia.

Korsakoff syndrome (KS) is a neuropsychiatric disorder that is caused by thiamine deficiency. Alcohol abuse accounts for 90% of thiamine deficiency [16,17], but other medical conditions can also lead to KS [18]. The syndrome is characterised by severe episodic memory deficits, which are mainly—but not exclusively—expressed as anterograde amnesia: the inability to encode and retrieve new memories. There is general consensus that these long-term declarative memory deficits are part of the cognitive profile of patients with KS [17]. While it was first assumed that working memory was largely spared (see review [19]), there is converging evidence suggesting that specific aspects of working memory (capacity) might be impaired in patients with KS [19,20,21,22]. As the previously mentioned studies used different outcome measures to estimate memory capacity, and the results show variability in the outcomes of memory capacity [19,20,21,22], straightforward interpretations of capacity scores are difficult. Furthermore, clinical observations point out that patients oftentimes show normal capacity scores when assessed in a test setting but encounter problems when memory is put to use in daily situations. So, rather than assessing *how much* information patients can possibly store, it could be of substantial value to assess *how* patients dynamically employ memory—reflected in their eye movement behaviour. Previous eye-tracking studies have already provided evidence that VWM usage is low when information is readily available in the outside world, but increases when sampling information becomes costly [9,10,11,12,13]. However, it is currently unclear what happens to the trade-off between sampling and storing when *storage* is more costly or diminished, i.e., in the case of memory deficits. Here, we investigate the tendency to sample externally versus storing internally in a copy task based on information availability *and* memory functioning. 

Participants were instructed to rebuild an example puzzle as fast and accurately as possible in an empty grid by dragging the pieces of the puzzle to the correct location. If the information-to-be-copied remained available in the outside world, we expected both individuals with and without memory impairments to heavily rely on external sampling. When the cost of sampling increased (i.e., information became less readily available), individuals without memory impairments were expected to shift their strategy towards memorising information [9,10,11,12,13,14]. Importantly, however, we expected patients with Korsakoff amnesia to adhere to the sampling strategy more than the healthy controls, because the cost of memorising as imposed by the individuals’ memory conditions outweighs the increased cost of sampling. Not only did we expect to find a different trade-off between groups, we also expected the degree of memory deficits to influence the trade-off: the more severe the memory deficit, the more heavily patients would need to rely on sampling over storing. With every extra item that can be memorised (i.e., span increase as measured in traditional memory tasks), people could theoretically load up an extra item per sample, and were therefore expected to rely less on sampling, particularly when information was not readily available and sampling was deemed to be costly. Regarding the type of memory deficits, we would specifically expect this hypothesis to hold for individuals with a higher capacity for traditional outcomes of *visual* working memory.

The current study aims to provide a first step in identifying eye movement markers indicative of subtle changes in memory usage that cannot be captured by means of assessing one’s maximum storage load, but that rather occur in dynamic interaction with our environment. 

## 2. Materials and Methods

### 2.1. Participants

Patients with Korsakoff syndrome (KS) were recruited via Slingedael Korsakoff Centre of Expertise (see Supplementary Figure S1 for a patient flow chart). All patients fulfilled the DSM-V criteria for alcohol-induced major neurocognitive disorder [23] and had an extensive history of alcoholism. All patients had severe thiamine deficiency (Wernicke encephalopathy) before the onset of KS. None of the patients were in the Wernicke encephalopathy phase at the moment of testing, and all were treated according to available guidelines prior to KS diagnosis. Next, age- and education-matched controls without memory impairments were recruited via various public and university platforms (e.g., Facebook, family members, university intranet, and community centres). 

We aimed to recruit 25 patients and 25 controls. This number was based upon previous studies, feasibility of including patients, and a power analysis. Previous studies have reported varying sample sizes ranging from 7 [9] to 72 [13]. The original trade-off effect has been observed in a group as small as 7 participants [9], and a previous study from our lab replicated the effect using eye-tracking with 12 participants [10]. As we expected larger variability in our patient group, we aimed to recruit at least double the amount of participants in both groups. Furthermore, recruiting 25 patients was regarded as feasible given the logistical challenges that come with testing in patient institutions. 

Eventually, we were able to include 24 patients (see Supplementary Figure S1 for a patient flow chart) and 27 controls. With the current sample size, for a one-tailed t-test with a power of 0.8, we should have been able to reliably detect effects with Cohen’s d = 0.74 [24]. Effects usually reported in copy task paradigms are similarly large [11,14]. Moreover, the linear mixed-effects models that we used have higher power than t-tests. Therefore, we were confident that our study would have large enough power.

All participants had to speak Dutch and gave written informed consent prior to the start of the experiment. Healthy controls were compensated for their participation with EUR 7 per hour paid in increments of 30 min, and received compensation for travel costs. Patients were not reimbursed for participation. 

The project was approved by the Faculty Ethics Review Board of the Faculty of Social and Behavioural Sciences at Utrecht University (protocol numbers 21-0076 and 21-0270) and the local science committee of Slingedael Korsakoff Centre of Expertise. Consent was obtained, and the protocol was carried out in accordance with the Declaration of Helsinki and Utrecht University’s and the Faculty Ethics Review Board’s requirements.

### 2.2. Measurements

#### 2.2.1. Experimental Computer Tasks

*Apparatus*. Experimental tasks were run on a Windows 10 Enterprise computer with an Intel Core i7-4790 CPU and 16 GB RAM, and displayed on a 27 inch LCD monitor at a resolution of 2560 × 1440 pixels at 100 Hz. Subjects were seated in a dimly lit room and placed their heads in a chinrest ~67.5 cm from the monitor. An EyeLink 1000 eye-tracker (SR Research Ltd., Ottawa, ON, Canada) was placed in front of the monitor and was used to track the eyes at a sample rate of 1 kHz. Calibration and validation were performed manually using a 9-point grid, attempting to achieve a calibration error of less than 2 degrees of visual angle (dva). 

*Copy task.* We used an adapted version of the copy task that was developed in our lab [10]. The aim of the task is to provoke a strategy switch in relying on visual working memory versus sampling information from the outside world. The experiment was programmed in Python 3.7 using the PyQt5 library [25] for visual presentation and mouse and keyboard interaction. PyGaze [26] was used to interact with the eye-tracker. 

**Figure 1:**
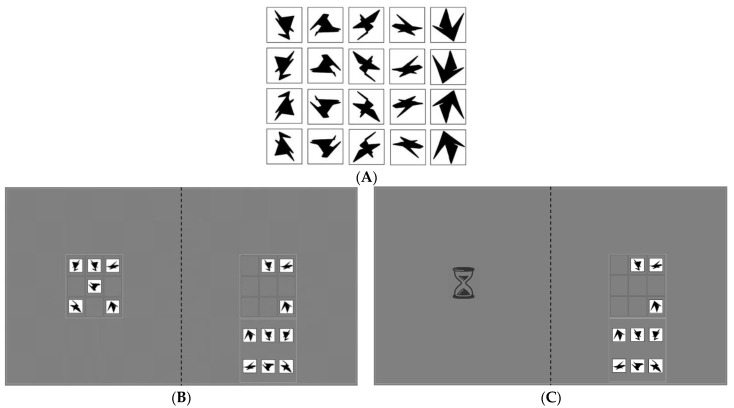
(**A**) All possible stimuli in the copy task. Adopted from [10,27]. An example trial is depicted for the baseline condition (**B**) and high-cost condition (**C**) of the copy task. On the left-hand side of the screen, the example grid was either visible or replaced by an hourglass for 2000 ms (i.e., gaze-contingent occlusion). On the right-hand side of the screen, the empty grid to place the items in (top) and the resource grid (bottom) were presented. A trial ended after 42 s. Note: the dotted midline is depicted for illustrative purposes and was not visible in the experiment.

Participants were instructed to copy a model puzzle of 6 items in a 3 × 3 ‘example’ grid on the left side of the screen to a 3 × 3 empty grid on the right side of the screen. Participants used a computer mouse with their preferred hand to drag one of the six items from the right bottom of the screen (the ‘resource’ grid) to the correct cell in the empty grid. The items were adopted from Arnoult [27] (Figure 1A) and consisted of black geometrical shapes that could not easily be named to measure reliance on VWM instead of verbalisation strategies [10]. 

The copy task consisted of two experimental conditions. In the baseline condition, the example grid was always visible (see Figure 1B). Therefore, the ‘cost’ to gather information from the outside world was low: information was freely available. In the experimental condition, a cost was introduced by manipulating when the example grid became visible. The example grid only appeared after fixating on the left side of the screen for a total of 2000 ms, during which an hourglass was presented (see Figure 1C). This ‘gaze-contingent waiting time’ was introduced to increase the cost associated with making an eye movement to sample information from the outside world.

Subjects were instructed to complete each puzzle as quickly and accurately as possible. Whenever an item was placed in the correct location, the background of the cell behind the item turned green for 700 ms and the item remained at that location. If the item was incorrectly placed, it disappeared from the location and the background of the cell turned red for 700 ms, after which subjects could make another attempt. After placing all six items correctly or after 42 s, the trial was ended. If all six items were placed correctly, positive feedback was shown. If subjects failed to correctly place all items within 42 s, a message appeared stating that they had ran out of time. By introducing a time limit, we aimed to urge subjects to adopt an efficient strategy [13]. 

The copy task was divided into two sessions, with each session consisting of two blocks. Subjects first performed three practice trials in the baseline condition to become acquainted with the task. Calibration and validation of the eye-tracker were performed after the practice trials. Then, in Session One, a baseline block of 15 trials was completed, followed by a high-cost block of 15 trials. In Session Two, again, a baseline block of 15 trials was completed, followed by a high-cost block of 15 trials, resulting in a total of 30 trials per condition. Although carry-over effects might have played a role [28], we deliberately chose a non-counterbalanced design a priori. The most important consideration was that the gaze-contingency in the high-cost condition was rather complex to understand, especially for patients. We deemed it more important that the basics of the task were understood first, only to introduce the gaze-contingent waiting time later on.

Before each trial, a drift check (max. 2 dva) was performed, and recalibration was performed when deemed necessary. After each block, subjects answered questions on their experience of commitment to and difficulty of the task (not considered in the current analysis). Each session of the copy task took 25–45 min, dependent on calibration time, task speed, and the number and length of breaks. 

First, we reported *completion time* and *number of correct placements,* descriptively. Only looking at the completion time would lead to a floor effect for participants who did not complete the trial within 42 s (i.e., some participants placed more items correctly than others), and only looking at the number of correct placements would lead to a ceiling effect for participants who completed the trial within 42 s (i.e., some participants were faster than others). We therefore calculated three performance measures in which the number of correct placements, total attempts (i.e., the sum of the number of correct and incorrect placements), and/or net copy time (i.e., completion time minus the waiting time for the hourglass) were taken into account. The *success rate* reflected the ratio between the number of correct placements and the total attempts. The *speed score* reflected the net copy time divided by the number of correct placements, that is, the net copy time per correctly placed item.

$$Success\;rate=\frac{Number\;of\;correct\;placements}{Total\;attempts}\;$$

$$Speed\;score=\frac{Net\;copying\;time}{Number\;of\;correct\;placements}$$

Second, eye movement outcomes of interest are listed below:

– *Number of crossings.* This refers to the count of only those saccades that crossed the midline from right to left, i.e., which jumped from the right (workspace) to the left of the screen (where the example puzzle was located). Crossings captured how often someone looked at the example over the course of the trial.
– *Dwell time per crossing.* This is the total duration of the fixations on the example divided by the number of crossings over the course of the trial. In other words, this score reflects how long someone viewed (i.e., encoded) the example per crossing.
– *Number of crossings per correct placement.* This refers to the count of only those saccades that crossed the midline from right to left, divided by the number of correct placements. This outcome expresses how often someone needed to inspect the model to place one item correctly.
– *Dwell time per correct placement.* This is the total duration of the fixations on the example, divided by the number of correct placements over the course of a trial. This reflects how much viewing time someone needed to place one item correctly.

Variables were aggregated per participant per condition by mean or median depending on the outcome measure (see Results).

Conceptually, a sampling strategy would translate to a relatively high number of crossings towards the example grid. A memorisation strategy would translate to a relatively low number of crossings towards the example grid. Memorisation is further expected to translate to longer dwell times per example grid visit to encode more items. 

We extracted various other variables (that were not included in the analysis, but serve a descriptive purpose) that can be found in the Supplementary Materials.

**Figure 2:**
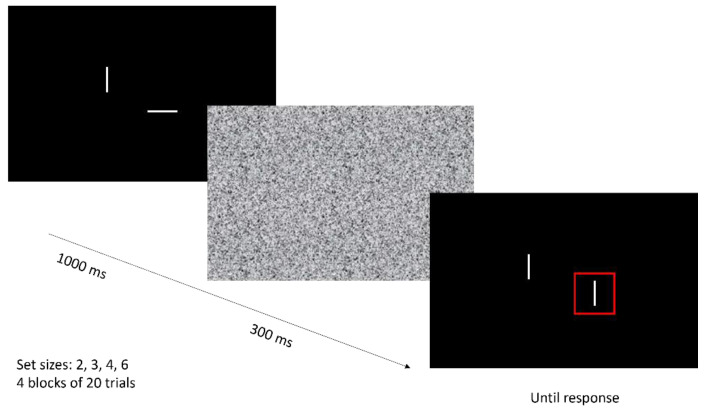
Trial example of a ‘change’ trial in the change detection task. Set sizes vary from 2 to 3, 4, or 6 white bars in orientations of 0°, 30°, 60°, 90°, 120°, and 150°. One of the bars is cued by a surrounding red square. Participants were instructed to indicate whether or not the orientation of the cued bar had changed.

*Change detection task.* Change detection tasks are often used in experimental research to assess working memory capacity [1]. Here, a simplified version of the paradigm from Luck and Vogel was used [20,29] (see Figure 2). With a varying set size of 2, 3, 4, or 6 items, white bars in different orientations (0°, 30°, 60°, 90°, 120°, and 150°) were presented on a black screen for 1000 ms, followed by a Gaussian random visual white noise mask for 300 ms. Consecutively, the bars were presented again. One bar was cued by a surrounding red square. The orientation of the cued bar changed in 50% of trials. The orientation of the non-target bars did not change. The participant was instructed to verbally report whether or not the orientation of the cued bar had changed. 

Five practice trials were completed. Here, subjects received feedback on their answers. After practising, 4 blocks of 20 trials each were presented. Every set size was presented 20 times in a random order. Here, subjects did not receive feedback on their answers. The task lasted for approximately 10 min. Eyes were not tracked; only behavioural responses were recorded. Kmax and *d*′ were calculated as outcome measures; Kmax is often used in the VWM literature [1,30] and allowed us to compare our findings with previous findings regarding patients with KS [20]. However, *d*′ is stated to yield a more robust outcome for visual working memory performance that is less prone to biases in response tendency than Kmax [31]. Therefore, we used *d*′ as the capacity score input in further analyses.

Kmax =(hit rate + correct rejection rate −1)× N (N = memory set size) 

$$d^{\prime }=z\left[p\left(\mathrm{hits}\right)\right]\;–\;z\left[p\left(\mathrm{falsealarms}\right)\right]$$

#### 2.2.2. Neuropsychological Tasks (See Supplementary Materials for Extensive Descriptions)

*Location learning task (LLT).* The standard stimulus set B of the modified location learning task (LLT) was used to assess visuospatial immediate and long-term recall [32,33]. The primary outcome measures were the learning index (amount of learning over five trials), placement errors (sum of errors over five trials), and the delayed recall score (subtraction of delayed recall placement error minus placement error of fifth trial). A negative score indicated a loss of information during the retention phase, whereas a positive score indicated better memory after the retention phase [33].

*Rey auditory verbal learning task.* The Rey auditory verbal learning task (RAVLT; 15 items, Dutch version [34,35]) was administered to assess verbal immediate and long-term recall. The outcome measures used were total number of correct words (range: 0–75) and number of correct words during the delayed recall (range: 0–15). Higher scores indicate better memory capacity.

*Digit span test (WAIS-IV).* We used the digit span subtest forward and backward from the Wechsler Adult Intelligence Scale—Fourth Edition (WAIS-IV [4]) to assess short-term auditory memory and verbal working memory. The longest sequence that was correctly repeated was used as an outcome measure for maximum capacity (range 2–8 or 2–9, for forward and backward, respectively). 

*Corsi block-tapping task.* The Corsi block-tapping task was used to assess visuospatial working memory [2,3]. We used a digitised version (thus, 2D) of the Corsi block-tapping task [36,37]. The forward subtest assesses short-term visuospatial attention; the backward subtest assesses VWM. The longest sequence that was correctly repeated was used as an outcome measure for maximum capacity (forward range 2–9; backward range 2–8). 

### 2.3. Procedure 

The test protocol (computer tasks + neuropsychological tasks) was administered with prioritisation of tasks with higher importance, while keeping fatigue and physical discomfort (e.g., by keeping the head in the chinrest) at a minimum and taking into account protocols for the delayed assessment of the LLT and RAVLT. 

For patients with KS, we divided the test battery into two sessions over separate days (ranging from 1 to 14 days apart, except for one patient who performed the Corsi block-tapping task in Session 2 and only after 1.5 months). Before the first session, we checked whether patients had already performed some of the neuropsychological tasks as part of standard care or another scientific study that was carried out within six months prior to the experiment. If that was the case, they were exempt from that task; previously reported scores on those tasks were used in order to prevent unnecessary work load and possible practice effects. Sessions were ended after a maximum of 75 min, or when patients became too tired.

For healthy controls, the test protocol was administered in a single visit. The first and second part of the experiment were separated by a break of 10–20 min. The total administration duration for controls was maximum 3 h. 

Task administration in Session 1 comprised (in this order) the following: LLT—direct recall, copy task—first session, LLT—delayed recall, digit span WAIS IV, and, if time allowed, a fixation and free viewing task (not taken into account in the current analysis) was also conducted. Task administration in Session 2 comprised (in this order) the following: RAVLT—direct recall, copy task—second session, RAVLT—delayed recall, Corsi block-tapping task, and, if time allowed, the change detection task was also conducted. See Supplementary Table S1 for overview of the test procedure and sessions for controls and patients.

### 2.4. Data Analysis

#### 2.4.1. Pre-Processing

Saccades, fixations, and timestamps were extracted using the EyeLink 1000 parser (default EyeLink saccade detection algorithm, SR Research Ltd., Canada). Data pre-processing was implemented using Python 3.10. Data analyses were conducted using R 4.1.2 [38]. 

#### 2.4.2. Demographics

Groups (i.e., controls and patients) were matched on age and level of education, since these factors are related to performance on memory tasks [39,40]. Mann–Whitney U tests were performed to assure similarity between groups in terms of age and education. A chi-squared test was performed to check sex distributions across groups.

#### 2.4.3. Dynamic VWM Strategy

To analyse differences in VWM strategy across conditions, and to assess whether individuals with memory impairments indeed adhered to the sampling strategy more than those without, we included all trials in a linear mixed-effect model (LMM [41]). This approach takes into account missing data and individual differences within groups. The LMM is robust against deviations from normality of the outcome variables [42]. Several linear mixed-effects models were generated to analyse the best fit for the data using the lmer function (lme4 package [43]) in R [38]. Factors included were Group, Condition, Group*Condition, and random slope and intercept for individuals. A likelihood ratio test (ANOVA function of the ltm package [44]) was used for model comparison to investigate which model outperformed the others in explaining the data (a lower AIC/BIC indicated a better fit). *χ*^2^ with α < 0.05 was fundamental in deciding on the most informative model. After fitting the model, the significance of factors was judged using a value of *p* < 0.05. The normality of the residuals was visually examined and confirmed for every linear mixed-effects model. Effect sizes were reported as standardised beta-coefficients (β) with a 95% confidence interval. The dependent variables were as follows: *success rate, speed, number of crossings*, *dwell time per crossing, number of crossings per correct placement*, and *dwell time per correct placement.* Given that we performed 18 tests (6 models × 3 factors), and used an alpha of 0.05, fewer than one of our findings is likely to be a false positive. This should be considered when interpreting the results. 

Datasets of all participants were analysed, initially without the removal of outliers. In addition, to make sure that findings were not driven by outliers, we removed those participants whose aggregated scores were ≥1.5 times the interquartile range apart from their group median for that specific outcome measure in that specific condition (baseline or high-cost). If participants were identified as an outlier in *one* condition, their data were removed from *both* conditions. After outlier removal, we ran the analyses again. Information on outliers is mentioned in the section of the respective analyses. 

#### 2.4.4. Memory Functioning and VWM Strategy

We expected that the degree and type of memory deficits *within our patient sample* would influence the trade-off between sampling and storing (e.g., lower capacity is expected to relate to more sampling). Therefore, we generated (non-parametric) regression models to predict the *number of crossings per correct placement* and *dwell time per correct placement* in both conditions as a function of memory capacity scores, given age and level of education. These outcome measures were chosen as they reflected both eye movement sampling behaviour and successful memory employment (‘per correct placement’). Each of the capacity scores was included in a separate regression model to predict behaviour in the copy task. We ran the models for both conditions separately, as we hypothesised that memory capacity would influence behaviour mostly in a situation where it is beneficial to tax working memory (high-cost condition) and not necessarily when information is freely available. 

We hypothesised that forward and backward span in the Corsi block-tapping task, forward and backward span in the digit span task, and sensitivity (*d*′) in the change detection task (see pre-registration on OSF: https://osf.io/83nsw/, accessed on 5 March 2023) would be related to sampling measures; for each outcome measure, higher scores were expected to result in fewer samples. Other memory task scores (LLT learning index and placement errors and RAVLT total score) were included in the pre-registration for exploratory purposes. Eventually, other than those pre-registered, we did not take all capacity measures into account. We decided to reduce the number of capacity measures, and with that the number of statistical tests, in order to prevent power issues. We decided to only look at the backward span, and not the forward span, of the Corsi block-tapping and digit span task; forward scores are clinically mostly interpreted as an (required) attentional span, whereas backward scores are taken as a working memory span. Furthermore, we decided to only analyse one outcome (instead of three) of the LLT (placement errors). 

In clinical neuropsychological practice, raw memory scores are corrected for age and level of education. As memory functioning is to some extend related to these variables [39], and these could confound the influence we attribute to (working) memory capacity scores, level of education and age were included as separate factors in each model. To correct for multiple testing, a Holm–Bonferroni correction was applied per condition (i.e., baseline, high-cost) and dependent variable of the copy task (i.e., crossings per correct placement, dwell time per correct placement). 

### 2.5. Code and Software

Experiment code, raw and pre-processed eye movement data, raw scores on neuropsychological assessment, and analysis scripts are publicly available and can be found at Open Science Framework: https://osf.io/83nsw/, accessed on 5 March 2023).

## 3. Results

### 3.1. Demographics

Thirty-two patients with Korsakoff syndrome (KS; 24 male, *M* = 63.5 years, *SD =* 7.56 years, range 47–76) were recruited via Slingedael Korsakoff inpatient Centre of Expertise. One patient dropped out after the introduction of the test session. One patient dropped out of the copy task after the practice session. Two patients were not able to complete the copy task (using a computer mouse) due to motoric impairment. We were unable to track the eyes of another three patients. After checking the medical file, one patient appeared to have suffered a partial stroke. Eventually, twenty-four patients were included (see Table 1 for demographic characteristics and see Supplementary Figure S1 for a patient flow chart). Patients were without known visual field deficits and had normal or corrected-to-normal vision, except for one patient who had retinal detachment of the left eye. Two patients could not perform the second test session; one deceased and one was bedridden. Due to lowered workload capacity, not all patients were able to complete all of the neuropsychological tasks in the available time.

A total of 27 controls (10 male, *M* = 58.48 years, *SD =* 8.86 years, range 40–81) were recruited to perform the same test protocol as the patients with KS. 

Table 1 shows group demographics, obtained scores on neuropsychological assessment, and statistical comparisons between groups. No significant differences between groups were found regarding age (U = 251, *p =* 0.171, *r* = −0.23). The level of education differed between groups, where healthy participants had a higher educational level (*M* = 5.9, *SD* = 0.92) than patients with KS (*M* = 4.46, *SD* = 1.14; U = 536.5, *p* < 0.001, *r* = 0.656). In both groups, however, the level of education was not significantly related to any of our copy task outcome measures in both conditions (all *p* > 0.064). For age, significant correlations were found in both conditions in both groups, but these effects were accounted for as the groups were age-matched. See Supplementary Table S2 for statistics on these correlations.

### 3.2. Dynamic VWM Strategy

#### 3.2.1. Data Loss

A total of 1440 trials were planned to be collected across 24 patients (2 sessions × 2 conditions × 15 trials). We removed every first trial in a block from the analysis: this trial served to check whether instructions were retained (additional encouragement was given when needed) and to habituate the participant to the new situation (e.g., transition to gaze-contingent block; −96 trials). Two patients did not complete the second session; one deceased and one was bedridden (−56 trials). Additionally, one patient was not able to finish the gaze-contingent condition in the first session due to a bug in the code (−14 trials). Any reason that could possibly interfere with performance (excessive movement of the participant, forgetfulness of task instructions, apathy, or problems controlling the mouse) was logged, and the corresponding trials (71 trials) were removed from further analysis. Eight trials were removed because the eye-tracker lost signal. To summarise, 245 trials were excluded, leaving 1195 trials for analysis. 

A total of 1620 trials were planned to be collected across 27 healthy controls (2 sessions × 2 conditions × 15 trials). Again, we removed every first trial in a block from analysis (−108). Due to, e.g., coaching or movement, four additional trials needed to be excluded from the analysis. Although a drift check was implemented, some trials had started with a drift check above the 2 dva threshold. If the error exceeded a 5 dva threshold, the trials were excluded to make sure that this would not confound our definition of a crossing (see Supplementary Materials for drift check descriptives). For one participant, this meant that almost none of the trials in the second session were valid. We therefore excluded the whole second session of this participant. In sum, we excluded 45 trials because of exceeding the drift check threshold. Finally, 1463 trials were left for analysis. 

Table 2 displays the outcomes of interest in the copy task for both groups, split per condition. Per participant, the outcome measures were aggregated by the mean over trials per condition, except for time-based outcome measures which were aggregated by the median. The group scores (i.e., medians) were then calculated. 

#### 3.2.2. Behavioural Performance

**Figure 3:**
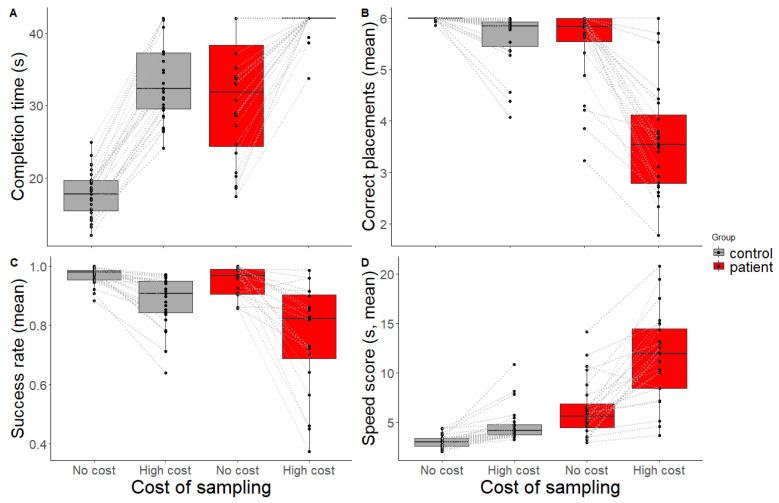
Performance scores. (**A**) Completion time (Mdn, 42s time limit). When introducing a high-cost gaze-contingent waiting time, patients failed to complete the puzzle within the time limit. (**B**) Mean correct placements (maximum 6) per trial, (**C**) mean success rate, and (**D**) mean speed score for controls (grey) and patients (red) across conditions (no-cost, high-cost). Black dots and grey lines represent scores for individual participants.

Completion time is depicted in Figure 3A. In the baseline condition, all controls and almost all patients were able to complete the trials within the time limit. When introducing the gaze-contingent waiting time in the high-cost condition, most of the controls were still able to complete the puzzle, but patients struggled to place all six items in the correct location within the time limit. 

Figure 3B shows the number of correct placements within a trial, which shows lower values for patients, especially in the high-cost condition. 

As a measure of how effective people were in placing items correctly without making errors, the *success rate* (i.e., the number of correct placements divided by the total number of attempts) was calculated (see Figure 3C). A linear mixed-effect model was fit to the *success rate* to analyse the influence of group and condition, while controlling for individual differences. There was no main effect of group (t = −1.53, *p =* 0.133, *β* = −0.05 [−0.12, 0.01]), but a main effect of condition (t = −3.37, *p =* 0.002, *β =* −0.24 [−0.38, −0.1]) was found, where the *success rate* was lower in the high-cost than the baseline condition. Additionally, there was an interaction effect between group and condition (t = −2.76, *p =* 0.009, *β =* −0.23 [−0.4, −0.07]), with patients performing disproportionately worse than controls in the high-cost condition. Two outliers (two healthy controls) were detected for *success rate*. After outlier exclusion, the linear mixed-effects model was run again. A main effect of group on success rate appeared (t = −2.12, *p =* 0.037, *β =* −0.07 [−0.13, −0.01]), where controls outperformed patients. The effects of condition and the interaction remained the same (see Supplementary Table S3 for results before and after outlier removal). 

*Speed score* is depicted in Figure 3D. A linear mixed-effect model was fit to *speed score* to analyse the influence of group and condition. The model showed a main effect of group (t = 5.57, *p* < 0.001, *β =* 0.03 [0.19, 0.4]), with patients being slower than controls. A main effect of condition was also present (t = 2.97, *p =* 0.005, *β* = 0.15 [0.05, 0.5]); participants took longer to place one item correctly in the high-cost condition compared to the baseline. In the high-cost condition, patients became disproportionately slower than controls as indicated by an interaction effect (t = 4.65, *p <* 0.001, *β =* 0.28 [0.16, 0.41]). 

For *speed score*, five outliers were detected (three healthy controls, two patients). After outlier removal, no differences were found in the results. See Supplementary Table S3 for statistical results before and after outlier removal. 

#### 3.2.3. Sampling Behaviour

The previous analyses showed that patients had more difficulty completing the task compared to controls: more mistakes were made and they were slower. So, how did participants arrive at their performance? The next question was whether or not patients showed the same eye movement behaviour as controls across conditions, and whether patients with memory impairment indeed adhered to a sampling strategy more than the controls. 

**Figure 4:**
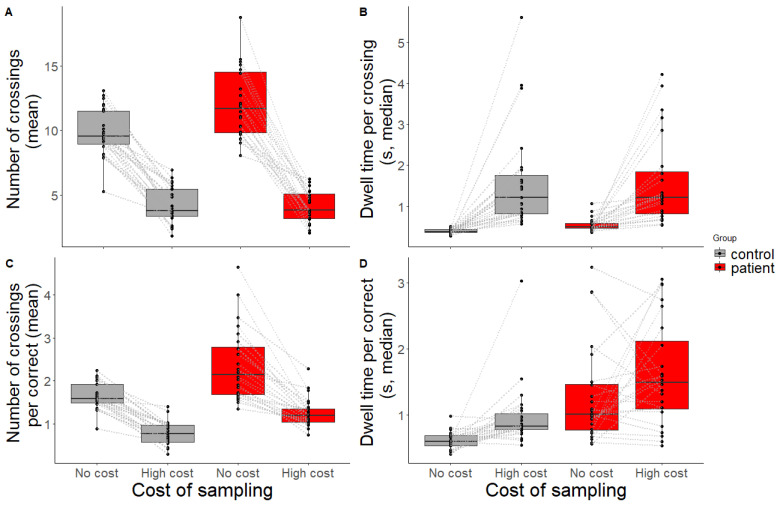
Eye movement measures as indicator for sampling behaviour. (**A**) Mean number of crossings within a trial, (**B**) median dwell time per crossing, (**C**) mean number of crossings needed to make one correct placement, and (**D**) median dwell time needed to make one correct placement for controls (grey) and patients (red) across conditions (no-cost, high-cost). Black dots and grey lines represent outcomes of individual participants.

Both the *number of crossings* (Figure 4A) and the *dwell time per crossing* (Figure 4B) were significantly predicted by group (*t =* 3.47, *p =* 0.001, *β =* 0.25 [0.11, 0.39]; *t =* 2.92, *p =* 0.004, *β* = 0.05 [0.02, 0.08], respectively) and condition (*t =* −12.1, *p* < 0.001, *β =* −0.65 [−0.75, −0.54]; *t =* 4.97, *p <* 0.001, *β =* 0.45 [0.27, 0.63], respectively). In general, patients sampled more and dwelled longer than controls. Both groups reduced sampling and dwelled longer when the sampling cost was high compared to when the sampling cost was low. An interaction effect was only found for the *number of crossings*: patients made fewer crossings in the high-cost condition (*t =* −3.44, *p =* 0.001, *β =* −0.22 [−0.34, −0.09]). This could (at least partly) be explained by the fact that they had more difficulty performing the task (being slower and less accurate, and therefore having less time within the trial to make a crossing). No outliers were detected for the *number of crossings*. Seven outliers were detected for the *dwell time per crossing* (three healthy controls, four patients). Then, the new fit yielded a main effect of group (t = 2.25, *p =* 0.025, *β =* 0.04 [0.01, 0.08]) and condition (t = 6.96, *p <* 0.001, *β =* 0.44 [0.32, 0.57]), which aligns with the findings before outlier removal. See Supplementary Table S3 for results before and after outlier removal. 

When looking at sampling behaviour with respect to *placing one item correctly* (Figure 4C,D), the same pattern was observed: patients made significantly more encoding crossings than controls (*t =* 4.08, *p <* 0.001, *β* = 0.38 [0.2, 0.56]) and both groups made fewer crossings in the high-cost condition compared to the no-cost condition (*t =* −8.65, *p <* 0.001, *β =* −0.47 [−0.57, −0.36]). Looking at the absolute values, the results show that the controls were able to retain multiple items per crossing (<1 crossing per correct placement) in the high-cost condition, whereas patients still needed one crossing or more. Patients also dwelled longer than controls to correctly place one item (*t =* 4.61, *p <* 0.001, *β =* 0.28 [0.16, 0.4]). In the high-cost condition, both groups dwelled longer to place one item correctly (*t =* 2.13, *p* = 0.039, *β* = 0.16 [0.01, 0.31]). No interaction effects were present for crossings per correct placement nor dwell time per correct placement (*t =* −1.59, *p* = 0.12, *β =* −0.1 [−0.23, 0.02], and *t =* 0.97, *p =* 0.338, *β =* 0.09 [−0.09, 0.26], respectively). For the *number of crossings per correct placement*, two outliers were detected (two patients). After the removal of the outliers, the same effects were found as before removal. Finally, for *dwell time per correct placement,* six outliers were detected (three healthy controls, three patients). The results of the new model fit show a main effect of group, but the effect of condition vanished: here, the effect of condition was driven by the outliers. Participants did not sample longer *per correctly placed item* in the high-cost condition. Nonetheless, the interaction effect held. Again, see Supplementary Table S3 for results before and after the outlier removal.

##### Note on Multiple Testing

We corrected for multiple testing by taking the least significant finding with a pinch of salt, as this might have reflected a false positive. This concerns the effect of condition found for *dwell time per correct placement* (*t =* 2.13, *p =* 0.039, *β* = 0.16 [0.01, 0.31]). 

### 3.3. Memory Functioning and Dynamic VWM Use

To explore whether the *degree* and *type* of memory deficits have an influence on sampling behaviour within the patient sample, regression models were generated to predict the *number of crossings per correct placement* and *dwell time per correct placement* in both conditions as a function of memory capacity scores, given age and level of education. Table 3 shows the regression estimates and uncorrected and Holm–Bonferroni-corrected (for variables of interest) *p*-values for each model.

Opposed to what was expected in the *baseline* condition specifically, the uncorrected raw *p*-values show that some of the capacity scores from traditional neuropsychological working memory assessment (digit span, Corsi) were related (with a medium effect size) to sampling behaviour. A higher capacity in the digit span yielded fewer crossings to place one item correctly (*p =* 0.034, *β* = −0.45 [−0.87, −0.04]). A higher capacity in the Corsi yielded fewer crossings (*p = 0*.019, *β* = −0.49 [−0.89, −0.09]) and shorter dwell times (*p* = 0.038, *β* = −0.41 [−0.8, −0.03]) to place one item correctly. So, the higher the memory capacity, the lower the number of crossings and the lower the dwell time that were needed to place one item correctly. 

In the condition with the gaze-contingent waiting time (high-cost condition), there was one predictor: *d*′. The higher the *d*′—indicating better visual working memory performance—the lower the number of crossings per correctly placed item (*p* = 0.023, *β* = −0.49 [−0.9, −0.08]), but the higher the dwell time (*p* = 0.011, *β* = 0.62 [0.17, 1.08]). Contrary to our expectations, there were no significant relations between the other memory capacity measures and sampling behaviour in the high-cost condition (all *p* > 0.351). 

After correcting for multiple comparisons, none of the relations in either the baseline or high-cost condition remained significant. Therefore, the general conclusion is that the degree and type of memory deficits did not predict sampling behaviour (sampling nor dwelling) in either of the conditions for the patients with KS. 

## 4. Discussion

In neuropsychological assessment of visual working memory (VWM), estimating the maximum capacity is currently the gold standard. However, previous studies have shown that, if possible, people prefer to fall back onto (i.e., sample from) information in the external world instead of memorising it [9,10,11,12,13]. Only when sampling is impeded do people decrease the amount of inspecting behaviour and instead memorise more information at once [9,10,11,12,13]. We hypothesised that when memory is impaired, an even more pronounced reliance on external sampling would occur. We assessed whether eye movements (used for external sampling) that are made during the execution of a memory task can serve as a proxy for VWM use in a group of healthy controls and patients with Korsakoff amnesia. 

Our dynamic working memory task yielded eye movement behaviour in healthy controls in line with the expectations: controls sampled often when possible, and sampled less often when information was less readily available. In the latter situation, they increased their dwell time on the model. This behaviour is in line with that observed in previous studies using a copy task that manipulated the availability of information [9,10,11,12,13,14]. It shows that whether or not information was available provoked different eye movement patterns in our healthy population.

Furthermore, our results indicate that patients with Korsakoff syndrome (KS) relied more on sampling—and thus on the external world as a memory buffer—than controls. This difference between groups was already observed when information was freely available in the external world. While executing our copy task, patients inspected the example on average 2.14 times to place one item correctly, whereas controls only looked 1.59 times. The values in our study indicate that both patients and controls inspected the example more than once in order to place one item correctly and thus often *re*inspected the example before making a placement. 

This reinspection behaviour conceptually replicates earlier findings, where results showed that people, when given the opportunity, will not load up more than roughly one item in VWM per inspection [10,14].

When information was less readily available in the external world (i.e., which we manipulated by introducing waiting time whenever the participant viewed the example), patients and controls adapted their behaviour: both groups sampled less often compared to when the information was freely available, but the encoding time per sample increased. We interpreted this as an attempt to memorise more information at once. Nonetheless, the waiting time, which induced the shift in strategy from sampling to memorisation, came at a cost. Participants made more errors and were slower. In patients with KS, this cost was the most profound: patients had difficulty completing the trial within the time and obtained lower performance scores than controls. So, although patients dynamically adapt their strategy when confronted with less accessible information—as reflected in their eye movement behaviour—they fail to do so as effectively as controls. Furthermore, in order to successfully place one item correctly, patients needed to sample more often (1.2 times) than controls (0.77 times). This aligns with the expectation that patients would adhere to a sampling strategy more than controls, even when sampling was costly. 

The increased reliance on the external world could be explained by deficits in working memory. Indeed, patients with KS performed worse than controls on all (but one) classical tasks that assessed memory subdomains, which confirms their impaired memory ability relative to controls and aligns with earlier findings of compromised (working) memory in patients with KS [19,20,22]. This supports the idea that impaired memory ability causes increased sampling: patients who have difficulty encoding or retrieving information need to sample multiple times (and, importantly, more often than controls) to strengthen the memory trace before being able to make a correct placement. It is therefore tempting to attribute a heavier reliance on external sampling to memory problems solely. However, if these memory problems were to underly sampling behaviour exclusively, we would expect individuals performing at the low end of the capacity spectrum to rely most strongly on the external world. Interestingly, however, we found that (lower) capacity scores on memory subdomains were not predictive of (lesser) sampling—and thus externalisation—behaviour in patients with KS. The absence of this correlation adds to the mixed findings regarding the relation between memory capacity and sampling behaviour, where some studies find correlations while others do not [6,45,46]). These inconsistencies might partly be explained by different approaches in the assessment of reliance on the external world, ranging from offering the possibility to directly sample from the external world, to demanding a more active and thought-through role of the participant (intended offloading, writing). Furthermore, previous studies [6,45,46] used a different operationalisation of working memory capacity. For example, Meyerhoff et al. [6] used the Corsi block-tapping task forward span to estimate VWM capacity. To be able to compare our results with those found in the study of Meyerhoff et al., we conducted additional analyses with the inclusion of the forward span (both verbal and visual, see Supplementary Table S4), which showed that the forward span did not predict sampling frequency or duration. Thus, in our population, objective outcomes of memory capacity did not relate to the frequency of sampling. It is possible, however, that this relation was not observed because there was no linear relationship. Theoretically, it could be the case that there is some sort of threshold of memory functioning that is needed to not heavily rely on sampling, and people will continuously sample when this threshold is not reached. Furthermore, the stimuli that are used to estimate capacity in traditional tasks have different visual features than the stimuli used in our copy task. Possibly, estimating capacity by means of memorising a sequence of the currently used stimuli would yield different results. Still, we argue that patients with KS *should* be able to load up at least two items at once: none of the patients in our sample had a capacity score < 2 on any of the classical neuropsychological tests. Yet, they sampled multiple times to correctly place one item, even when the sampling costs were high. This argues against the idea that mere memory ability is at the core of sampling behaviour. If not ability, what then causes these heightened levels of sampling—both when information is freely available and when it is not—in patients compared to controls? 

The fact that we did not find a relation between the currently administered memory capacity tasks and sampling behaviour in our copy task could be because these tasks might measure different constructs of memory. Earlier studies that adopted copying tasks interpreted frequent external sampling as putting little reliance on internal VWM [9,10,11]. *Re*visits (sampling more than once per correctly placed item), subsequently, could then be interpreted as an expression of non-successful encoding upon the first inspection. However, recently, it was found that (re)visiting behaviour does not necessarily mean that VWM content is completely put to use before taking another look at the example [14]. Rather, it could be argued that sampling behaviour serves some sort of soothing behaviour to increase one’s confidence in their memory strength. This idea would fit with Morrison and Richmond [45], who suggested that the subjective estimation of one’s memory capacity influences sampling behaviour to a larger extent than objective memory capacity. The findings of both Sahakian et al. [14] and Morrison and Richmond [45] point out that the frequency of sampling is not inherently a proxy for the *amount* of information that is stored in VWM, which would explain why pure capacity scores are not predictive of the amount of sampling. 

Plausibly, sampling behaviour does not reflect the (in)ability to use memory, but reluctance to use memory as a consequence of higher costs to internally store information. With impaired memory, internally storing information, even for only one or two items, is likely associated with high effort, and sampling would be regarded as a more cost-efficient strategy even when sampling costs were large. With non-impaired memory, the effort associated with retaining multiple items per sample would be lower, and internally storing information would be regarded as the more cost-efficient strategy when sampling costs became large. In a healthy population, choosing externalisation over internal storage has been found to indeed depend on a perceived reduction in effort [46]. Offloading (in this case, writing down a sequence of letters) was perceived as requiring higher effort than internal storage for small set sizes. This pattern flipped with increasing set sizes [46]. Observations in our healthy population can therefore be aligned with the idea of reducing perceived effort: when we introduced the waiting time, sampling might have been perceived as being more effortful by controls than memorising a small number of shapes. For patients with KS, the increased cost of sampling did potentially not outweigh the cost associated with memorisation. Therefore, heightened sampling could be a reflection of increased reluctance to use internal memory storage in order to minimise perceived effort in patients with KS.

The decision to offload or memorise is not only dependent on effort, but also on the desire to be accurate: in a previous study where accuracy was at stake, participants were more inclined to fall back onto the external world to support memory, even when this would not necessarily lead to better performance compared to using only memory [46]. Sampling (here, *re*inspecting the example) could in this case be seen as an expression of checking behaviour. Our participants were instructed to perform the task as accurately and quickly as possible, but they were not punished for errors nor slowness other than receiving feedback. Errorless performance was therefore possibly not deemed to be as important. When checking was easy, people tended to revisit the example (>1 sample per correctly placed item), but when sampling was impeded, checking—and thereby assuring accuracy—might not have been worthwhile anymore. Actually, when sampling costs increased, it could be seen as a strategy shift to make more attempts (albeit faulty) to avoid sampling, and to ‘squeeze’ out more information from memory at the expense of accuracy [14]. Thus, sampling behaviour can vary depending on whether effort minimisation or time accuracy expenditure is prioritised. 

So, sampling behaviour is likely to be the end-product of (perceived) working memory ability [45], effort minimisation [46], and task demands (speed and/or accuracy) [14,46]. Additionally, our copy task did probably not only tax working memory in order to complete the puzzle as fast and accurately as possible; our task called upon a certain level of executive functioning to monitor what puzzle pieces had already been placed and to keep a structured workflow. Note that participants exerted control over the visibility of information: a gaze-contingent waiting time required them to wait for 2 s, after which they could decide how long they would inspect—and thus encode information from—the example puzzle. During the experiment, we observed that patients with KS needed more guidance in the task instructions. We suspect that some patients had difficulty understanding how to exert control over the gaze-contingent appearance of the example puzzle. This would fit with the frequent report of executive deficits in patients with KS [47,48]. Potentially, patients may have wanted to sample from the example more frequently, but lacked the full understanding of how to accomplish this. Indeed, our data (see Supplementary Figure S3) show that patients actually moved their eyes towards the side of the screen with the example more often, but only a part of these crossings remained fixated long enough to reveal the example puzzle. Then, when patients finally waited long enough to make the example appear, they could have been inclined to directly place the stimulus they encoded, failing to oversee the consequence of having to wait again to make the example reappear. This somewhat impulsive eye movement behaviour can be supported by the fact that disinhibitory control is often observed in patients with KS [49,50]. In the acute phase of Wernicke’s encephalopathy, which precedes the development of Korsakoff syndrome, oculomotor symptoms such as nystagmus are often observed (Wernicke, 1981, in [17]) and some of these may remain present in the chronic phase. Yet, as the outcome measures we used were rather crude, we do not believe that these would be influenced by nystagmus. Patients with KS display only subtle impairments in recognising and naming real world objects (letters) with degraded perceptual clarity or common objects (e.g., animals) from atypical perspectives [51]. Additionally, spatial perception is not hampered [51]. Furthermore, if patients would have had difficulty with perceiving the stimuli, they would have already performed worse in the baseline condition and differences between conditions could not have been explained by it.

Although a reduced understanding of task instructions could partly explain our results, we are confident that patients clearly understood the task manipulation. We base this upon the observation that they did perform a strategy shift: patients either decreased the amount of sampling and memorised more, or made more placement attempts (albeit faulty) in order to avoid sampling. Still, executive deficits could have contributed to their impaired performance on the task. For example, cognitive flexibility is associated with better performance on jigsaw puzzles [52], which are to some extent similar to our copy task. Patients with KS have shown deficits in this cognitive domain, where they are slower when rule switching is required, are worse at the inhibition of previously learned rules, and show more perseveration errors [53]). Likewise, it is possible that patients had more difficulty switching between blocks, that they stuck to their previous sampling strategy, and/or that they made perseveration errors in our task (e.g., placing the same stimulus at the same wrong location multiple times), leading to worse performance. As we do not have quantitative neuropsychological data on these functions, we cannot rule out the possibility that they influenced our results. Additionally, differences in psychomotor and information processing speed [52,54], and apathy [55] or other clinical manifestations of the syndrome such as depression [49,56], can partly explain the finding that patients took longer than controls. We acknowledge that there are multiple facets that may influence behaviour in our task. Despite the difficulty in disentangling the factors that contribute to visual working memory usage, we argue that exactly because of this, our task approaches more naturalistic VWM usage than mere memory tasks. After all, in daily life, the way that we deal with information is also the result of the complex interplay between several cognitive factors and the task at hand.

Although it is too early to directly translate our findings to a clinical implementation, we can speculate about the potential clinical value of a dynamic task such as ours. Diagnostically, a task such as ours offers the possibility to detect differences in working memory usage in a more dynamic environment than the classical working memory paradigms. It might allow one to reveal the different strategies that are put to use, and facilitate the detection of the switching abilities of the patient. Future research should elucidate how eye movement markers in these dynamic tasks predict functioning in (instrumental) activities of daily living. Once established, this could give insights into the extent to which patients are able to function independently, which might help in assigning patients to the care facility that is most adapted to their level of functioning.

Patients with KS often reside in clinical institutions that are tailored to the needs of this population [17]. One aspect that puts a burden on caretakers is the need to constantly remind patients with KS of important appointments or agreements, such as taking one’s medicines. To enlighten this burden, rehabilitation implementations revolve around finding solutions that fit patients’ memory functioning. Using ‘external memory’ in the form of notebooks or calendars has been described as among the most common in supporting people with memory deficits [57]. More specific to patients with KS, errorless and/or procedural learning in (instrumental) activities of daily living were investigated as novel approaches, as implicit memory is relatively spared in KS [58,59]. Other developments are aimed at using technologies to support memory in patients with KS [60,61]. With regard to these memory aids, a future direction might be to assess whether patients’ inclination to rely on the outside world is linked to the ability to effectively use these memory aids, e.g., whether patients benefit from sampling from a smartwatch (which is constantly available around the wrist) versus sampling from a notebook (which is not always in the same room as the patient). 

To conclude, our results offer a framework to think more thoroughly about how dynamic tasks such as ours could be used to combine diagnostics and rehabilitation.

*Limitations.* Several limitations need to be considered when interpreting our results and making future recommendations. The dynamic nature of the task resulted in higher complexity compared to neuropsychological tasks targeting cognitive functions in isolation, thereby involving other cognitive functions apart from working memory (see also [52]). We specifically designed the test battery to obtain an as broad as possible memory profile, but this came at the (foreseen) cost of excluding measures for other cognitive domains. Although we addressed how other cognitive factors might have potentially influenced our results, we could not substantiate these using objective measures. 

Second, we performed multiple analyses on the relation between capacity scores and sampling behaviour. No effects were present after correcting for multiple statistical tests. This might have been due to limited power, which could be resolved in the future by including larger sample sizes, or by reducing the amount of statistical tests by using, for example, compound scores for memory functioning or sampling behaviour. 

Furthermore, our experimental paradigm comes with several practical limitations. The requirement to use a computer mouse excluded severely motorically impaired patients from participating, and might have led to slower performance in participants who had little experience in using a computer mouse. Furthermore, using an eye-tracker in patient populations comes with general limitations relating to the inability to hold a position for an extended period, oculomotor deficits and/or droopy eyelids, the tendency to move the head, reinstating calibration and validation cycles, and so on. This could have led to an inclusion bias (e.g., non-compliant patients could not be calibrated or produced datasets with signal loss, and could therefore not be included). 

Although we successfully gathered eye-tracking data for our study, the technical challenges that come with eye-tracking should definitely be taken into account when using such tasks in clinical settings. To be able to use it for diagnostical and rehabilitation purposes requires profound knowledge of the apparatus and familiarity with the type of data. While eye-tracking paradigms can yield rich datasets and valuable knowledge, they should be finetuned to the patient population, and task administration time, exclusion criteria for participation and prospected outcomes should be weighed against the investment to implement such paradigms. Paradigms that do not involve eye-tracking but measure sampling differently (e.g., by mouse movements such as in [6,14]) might offer solace, although future research should elucidate whether and how these different outcome measures (hand vs. eye movements) are directly interchangeable. 

## 5. Conclusions

Differences in performance and sampling behaviour between patients with KS and healthy controls could be driven by several factors. Although we cannot (yet) pinpoint the (most pronounced) underlying factor causing sampling behaviour, assessing sampling behaviour clearly yields additional value on a clinical level as to *how* patients dynamically use information in situations that demand memory usage. We conclude that Korsakoff amnesia evokes a relatively heavy reliance on external sampling, even when sampling is costly. Naturalistic eye movement markers can serve as a proxy for these subtle changes in memory usage that are not captured by assessing one’s maximum storage capacity, but that rather occur in dynamic interaction with the environment.

## Figures and Tables

**Table 1:** Demographic characteristics and scores on the neuropsychological memory tasks per group.

	Patients with KS			Healthy Controls			Test Statistic ^a^		
	*n*	Mdn (IQR)	Range	*n*	Mdn (IQR)	Range	χ^2^	*p*	*d*
**Demographics**									
Sex	24	16 male		27	10 male		3.357	0.067	0.53
							**U**	***p***	**r**
Age, years	24	64 (8.5)	47–74	27	59 (8.5)	40–81	251	0.170	−0.23
Level of education	24	4.5 (1.25)	3–7	27	6 (2)	4–7	536	<0.001 **	0.66
Time since admission, years	24	3.1 (7.4)	0.1–16.9						
**Neuropsychological task scores**									
Location learning task	23			27					
Total displacement score		85.0 (50.5)	45–129		31 (25.5)	3–75	28	<0.0001 ***	−0.91
Learning index (0–1)		0.11 (0.08)	0.03–0.3		0.53 (0.4)	0.1–1	582	<0.0001 ***	0.87
Rey auditory–verbal learning task	22			27					
Immediate recall: Total correct (0–75)		25 (7.5)	14–36		47 (17)	33–67	584	<0.0001 ***	0.97
Delayed recall: Total correct (0–15)		1 (2)	0–4		9 (6)	3–14	590	<0.0001 ***	0.99
Digit span test (WAIS-IV)	24			27					
Forward span (2–9)		5 (1)	4–8		6 (1.5)	4–9	458	<0.01 *	0.42
Backward span (2–8)		4 (2)	2–6		5 (1.5)	2–8	483	<0.005 **	0.49
Corsi block-tapping task	23			27					
Forward span (2–9)		5 (0)	1–8		5 (1)	3–8	394	0.076	0.27
Backward span (2–8)		5 (1)	2–7		6 (1)	2–7	449	0.005 **	0.45
Change detection paradigm	19			27					
Average K_max_ score		1.21 (0.67)	0.59–1.93		2.17 (0.79)	0.43–3.45	450	<0.0001 ***	0.75
D-prime		1.29 (0.45)	0.82–1.99		2.27 (0.64)	0.63–3.8	456	<0.0001 ***	0.78

Sample size *n*, median Mdn, interquartile range IQR, range (min.–max.). ^a^ Non-parametric test statistics indicating group differences and effect sizes: chi-squared, *p*-value, and *d* for binomial variable sex, or Mann–Whitney–Wilcoxon U, *p*-value, and rank-biserial correlation *r*. * *p ≤* 0.05, ** *p ≤* 0.005, *** *p ≤* 0.0001.

**Table 2:** Outcomes of the copy task for patients with KS and healthy controls split by conditions (baseline, high-cost).

Copy Task Scores	Patients with KS			Healthy Controls		
	*n*	Mdn (IQR)	Range	*n*	Mdn (IQR)	Range
Completion time, s	24			27		
Baseline		31.85 (13.97)	17.46–42		17.75 (4.2)	12.1–24.95
High-cost		42 (0.001)	33.68–42		32.33 (7.75)	24.05–42
Net copying time, s	24			27		
Baseline		31.85 (13.97)	17.46–42		17.75 (4.2)	12.1–24.95
High-cost		32.34 (4.96)	21.65–37.77		24 (4.66)	18.54–36
Correct placements	24			27		
Baseline		5.84 (0.45)	3.22–6		6 (0)	5.89–6
High-cost		3.54 (1.33)	1.78–6		5.85 (0.47)	4.07–6
Success rate	20			25		
Baseline		0.97 (0.08)	0.86–1		0.97 (0.03)	0.88–1
High-cost		0.82 (0.22)	0.37–0.99		0.91 (0.11)	0.64–0.97
Speed score, s	24			27		
Baseline		5.622 (2.4)	3–14.17		3.04 (0.77)	2.1–4.41
High-cost		11.95 (6.02)	3.68–20.77		4.15 (0.99)	3.28–10.84
Number of crossings	24			27		
Baseline		11.7 (4.68)	8.07–18.78		9.54 (2.57)	5.29–13.07
High-cost		3.82 (1.9)	2.04–6.3		3.79 (2.12)	1.82–6.96
Dwell time per crossing, s	24			27		
Baseline		0.49 (0.12)	3.56–1.06		0.38 (0.08)	0.28–0.51
High-cost		1.22 (1.03)	0.54–4.22		1.21 (0.94)	0.56–5.62
Number of crossings per correct placement	24			27		
Baseline		2.14 (1.1)	1.35–4.65		1.59 (0.43)	0.88–2.24
High-cost		1.2 (0.31)	0.75–2.29		0.77 (0.4)	0.30–1.4
Dwell time per correct placement, s	24			27		
Baseline		1.01 (0.69)	0.56–3.23		0.60 (0.16)	0.41–0.98
High-cost		1.49 (1.03)	0.54–3.05		0.83 (0.24)	0.55–3.03

Valid datasets *n*, median Mdn, interquartile range (IQR), and range (min.–max.).

**Table 3:** Unstandardised coefficient estimates, raw *p*-values, and Holm–Bonferroni-adjusted *p*-values for factors (fixed covariate levels of education and age, and capacity score of interest) within the regression models to predict sampling behaviour of the patients with Korsakoff syndrome in the copy task (crossings per correct placement, dwell time per correct placement) split by condition (baseline, high-cost).

	No. of Crossings per Correct Placement						Dwell Time per Correct Placement					
	Baseline			High-Cost			Baseline			High-Cost		
	Est.	Raw *p*	Holm	Est.	Raw *p*	Holm	Est.	Raw *p*	Holm	Est.	Raw *p*	Holm
**Digit span—BW span**												
*N = 24*												
*Education*	−0.042	0.782		0.059	0.377		0.087	0.506		0.120	0.464	
*Age*	0.026	0.298		0.014	0.215		0.035	0.106		−0.031	0.251	
*Digit span backward*	−0.349	0.034 *	0.306	−0.028	0.681	1	−0.261	0.061	0.427	−0.033	0.845	1
**Corsi—BW span**												
*N = 23*												
*Education*	−0.127	0.413		0.053	0.445		0.015	0.911		0.093	0.588	
*Age*	0.03	0.234		0.013	0.249		0.039	0.077		−0.029	0.293	
*Corsi span backward*	−0.402	0.019 *	0.19	−0.061	0.393	1	−0.300	0.038 *	0.306	−0.043	0.808	1
**D′**												
*N = 19*												
*Education*	−0.165	0.440		−0.065	0.190		0.053	0.681		0.088	0.609	
*Age*	0.027	0.394		0.017	0.030 *		0.034	0.085		−0.009	0.724	
*D*′	−0.643	0.338	1	−0.377	0.023 *	0.207	−0.399	0.334	1	1.541	0.011 *	0.11
**LLT—Placement errors**												
*N = 23*												
*Education*	−0.072	0.664		0.044	0.516		0.079	0.564		0.11	0.503	
*Age*	0.028	0.341		0.018	0.134		0.031	0.203		−0.026	0.372	
*Placement errors*	−0.000021	0.998	1	0.001	0.744	1	−0.003	0.638	1	0.006	0.351	1
**RAVLT—Total score**												
*N = 22*												
*Education*	−0.070	0.714		0.024	0.756		0.025	0.875		0.046	0.809	
*Age*	0.027	0.424		0.022	0.115		0.043	0.119		−0.018	0.585	
*Total score*	−0.007	0.843	1	0.009	0.500	1	0.02	0.483	1	0.03	0.386	1

Notes: digit span—BW span = backward span in the WAIS IV digit span, Corsi—BW span = backward span in the Corsi block-tapping test, D′ = *d-prime* in the change detection task, LLT—placement errors = total number of placement errors in the location learning task, RAVLT—total score = number of correctly reproduced words over five trials from the Rey auditory verbal learning task. * p < 0.05.

## Data Availability

The data presented in this study are openly available in Open Science Framework at https://osf.io/83nsw, accessed on 5 March 2023.

## Supplementary Materials

The following supporting information can be downloaded at https://www.mdpi.com/article/10.3390/jcm12113630/s1. Additional descriptions, tables, figures, and analyses as indicated in the report can be found in the Supplementary Materials.
