# Supplementary material for: Eye Movements as Proxy for Visual Working Memory Usage: Increased Reliance on the External World in Korsakoff Syndrome
Source: J Clin Med. 2023 May 23;12(11):3630. doi: 10.3390/jcm12113630 (PMC10253737; doi:10.3390/jcm12113630)
Supplement: Supplementary file 1 [file jcm-12-03630-s001.zip › jcm-2305842-supplementary.pdf]

Supplementary Materials

Methods.....2
Participants.....2
Patient flow chart.....2
Measurements.....3
Experimental computer tasks......3
Neuropsychological tasks – Extensive task descriptions.....3
Procedure – session division for controls and patients.....5
Results.....6
Correlations with Copy Task outcome measures.....6
Dynamic VWM strategy.....7
Data loss – drift check descriptives......7
Outlier removal.....8
Discussion.....9
Forward Span analysis.....9
Ineffective crossings.....10
References.....11

Methods

Participants

Patient flow chart

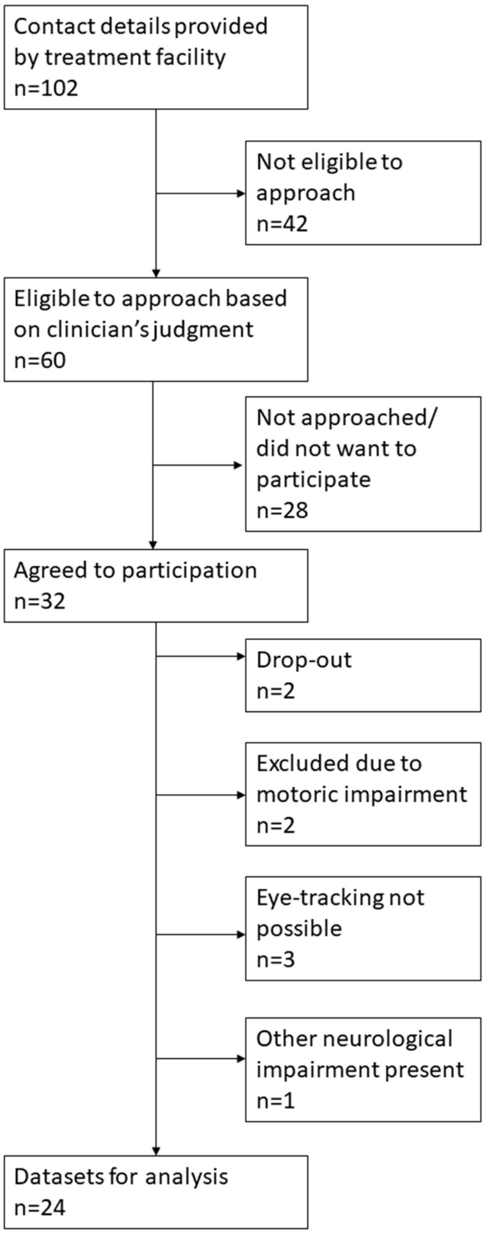

Figure S1. Patient flow chart of the recruitment and inclusion process.

Measurements

Experimental computer tasks.

Copy task – outcome variables. We extracted and processed various key variables from both conditions in the Copy Task. (1) *Completion time (s):* the median time (in seconds) someone took to finish the puzzle, or to reach the time limit. (2) The *net copying* time (s): the median time (in seconds) someone was actively copying, which is the completion time minus the waiting time for the hourglass. (3) The *number of crossings*: the mean count of how often someone inspected the example puzzle per puzzle. (4) The *dwell time at the model (s):* the median total time per trial that someone inspected the example puzzle in seconds. (5) The mean *number of correct placements*. (6) The mean *number of incorrect placements*. (7) The w*rong per correct* ratio: the mean ratio of how many mistakes someone made before placing a stimulus at the right location. (8) The number of c*rossings per correct item*: the mean ratio of how often the participant inspected the example puzzle to place one stimulus correctly. (9) The *dwell time at model per correct item (s):* the median time it took someone to encode enough information from the example puzzle to place one stimulus correctly in seconds (waiting time not included).

Neuropsychological tasks – Extensive task descriptions

Location Learning Task (LLT). Standard stimulus set B of the modified Location Learning Task (mLLT; [1,2] was used to assess visuospatial immediate and long-term recall. Subjects were given the instruction to closely inspect a board with a 5 x 5 matrix containing 10 pictures of objects for 15 seconds, and to memorize the locations of the objects as accurately as possible. This procedure was repeated for five times and after each presentation patients were instructed to place the items on the correct position (the correct cell) in an empty matrix. The ten object cards were given one by one in random order. Before the start, one practice trial (2 x 2 matrix containing two items) was performed to ensure task comprehension. After a delay phase (ideally 20-30 minutes, but due to various reasons in our sample ranging from 25 to 50 minutes), patients were unexpectedly asked to locate the objects again without seeing the stimulus board.

Primary outcomes measures are the learning index (amount of learning over five trials), placement errors (sum of errors over five trials), and the delayed recall score (subtraction of delayed recall placement error minus placement error of fifth trial). A negative score indicates

loss of information during retention phase, whereas a positive score indicates a better memory after the retention phase [2].

Rey Auditory Verbal Learning Task (RAVLT). The Rey Auditory Verbal Learning Task (RAVLT; 15 items, Dutch version; [3,4] was administered to assess verbal immediate and long-term recall. Participants were instructed to memorize a long list of words, without time or order restrictions. Fifteen unrelated, but easy to visualize words (subtest A) were read out loud (1 word every 2 seconds). The procedure was repeated five times. After each repetition, participants needed to recall all the words they memorized, also the ones that they mentioned in a previous trial. After a delay phase (ideally 20-30 minutes, but due to various reasons in our sample ranging from 25 to 50 minutes), patients were unexpectedly asked to recall the words again without hearing them again. Outcome measure used are: total number of correct words (range: 0-75) and number of correct words during the delayed recall (range: 0-15). Higher scores indicates better memory capacity.

Digit Span Test (WAIS-IV). We used the Digit Span subtest Forward and Backward from the Wechsler Adult Intelligence Scale – Fourth Edition (WAIS-IV; [5] to assess verbal working memory. The test administrator reads out loud a sequence of digits. Each part consists of eight items of each two series, that increase in length up to a maximum of 8 (backward) or 9 (forward) digits. During the DSTF, short-term auditory memory is measured, and the participant has to repeat the sequence in the same order. During the DSTB, the participant has to repeat the items backward to measure verbal working memory. The longest sequence that was correctly repeated was used as an outcome measure for maximum capacity (range 2–8 or 2-9).

Corsi Block Tapping Task. As a counterpart for the Digit Span test, the Corsi Block Tapping Task was used to assess visuospatial working memory [6,7]. We used a digitized version (thus, 2D) of the Corsi, where nine blue squares (30x30mm) were presented on a tablet (255x205 mm). A sequence of squares, that increases in length up to a maximum of 8 (backward) or 9 (forward), lights up in yellow (500ms flashing time, 1000ms interval, [8,9]. Participants were instructed to tap the squares in the same sequence or to tap them backwards. The forward subtest assesses short-term visuospatial memory; the backward

subtests assesses visuospatial working memory. The longest sequence that was correctly repeated was used as an outcome measure for maximum capacity (forward range 2–9, backward range 2-8), and total scores were calculated by multiplying this capacity score with the series-score (e.g., to gain insight in whether people only had one or two sequences correct for that span).

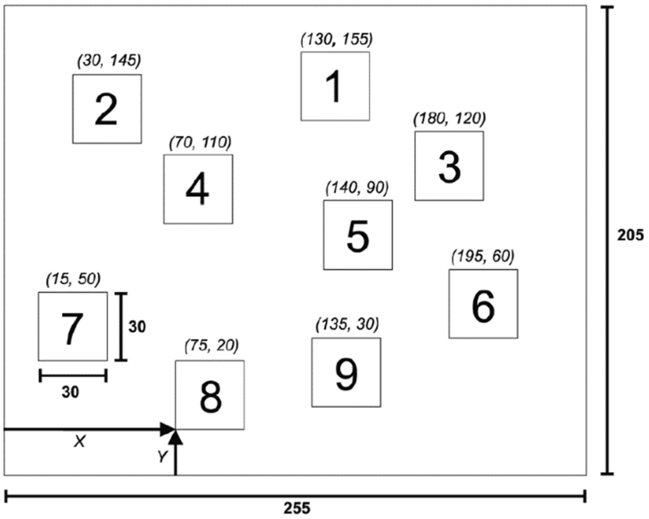

Figure S2. Layout of the tablet version of the Corsi Block Tapping Task. Adapted from [7].

Procedure – session division for controls and patients

Table S1. *Test procedure for healthy controls and patients with KS.*

	Day 1			Day 2
	Session 1		Session 2	Session 2
Healthy controls	1. LLT<br>2. Copy Task session 1<br>3. LLT – delayed<br>4. Digit Span WAIS IV<br><br>If time allowed:<br>Fixation and Free viewing	break	1. RAVLT<br>2. Copy Task session 2<br>3. RAVLT – delayed<br>4. Corsi Block Tapping Task<br>If time allowed:<br>Change Detection Task	n.a.
Patients with KS	1. LLT<br>2. Copy Task session 1<br>3. LLT – delayed<br>4. Digit Span WAIS IV<br><br>If time allowed:<br>Fixation and Free viewing	n.a.		1. RAVLT<br>2. Copy Task session 2<br>3. RAVLT – delayed<br>4. Corsi Block Tapping Task<br>If time allowed:<br>Change Detection Task

Results

Correlations with Copy Task outcome measures

Table S2. *Spearman's correlations (ρ, raw* p*-value) for correlations of educational level and age with the outcome measures on the Copy Task that are used in our prediction models for patients with Korsakoff's syndrome (KS) and healthy controls in both conditions of the Copy Task (baseline, high cost). Significant correlations were found for age and both performance and eye-movement measures on the Copy Task in both healthy controls and patients, but these effects are not bothersome in further group comparisons, as groups were age-matched.*

	**Baseline**				**High cost**			
	**Patients with KS**		**Healthy controls**		**Patients with KS**		**Healthy controls**	
	ρ	*p*	ρ	*p*	ρ	*p*	ρ	*p*
**Correlated variable: Educational level**								
Success rate	0.073	.756	0.270	.191	0.14	.557	0.089	.673
Speed score	0.106	.623	-0.34	.083	0.059	.786	-0.191	.341
Number of crossings	-0.187	.384	-0.094	.642	0.17	.428	-0.240	.227
Dwell time per crossing	0.384	.064	-0.115	.569	-0.108	.612	0.192	.337
Number of crossings per correct	-0.015	.945	-0.96	.632	0.205	.338	-0.323	.1
Encoding time per crossing per correct	0.136	.526	-0.105	.601	0.1	.64	0.117	.56
**Correlated variable: Age**								
Success rate	-0.084	.726	-0.3	.146	0.541	.014*	-0.072	.733
Speed score	0.362	.082	0.533	.004**	-0.153	.476	0.197	.325
Number of crossings	0.112	.591	0.125	.536	0.43	.036*	0.554	.003**
Dwell time per crossing	0.543	.006**	0.453	.018*	-0.399	.054	-0.5	.008**
Number of crossings per correct	0.216	.312	0.148	.461	0.462	.023*	0.573	.002**
Encoding time per crossing per correct	0.403	.051	0.313	.111	-0.26	.223	-0.212	.289

*$p \leq .05$, **$p \leq .01$, ***$p \leq .001$

Dynamic VWM strategy

Data loss – drift check descriptives.

A drift check was performed before the start of each trial, by computing the root mean squared error (RMS) of the gaze prediction on a central fixation cross which was shown for two seconds. When the RMS was greater than > 2° (degree visual angle), a warning message was displayed. Large RMSs could occur due to the participant not paying attention, not fixating stably, intermittent head movements during the trial due to which participants' position was changed, or drift of the eye tracker. Whenever a warning message was displayed, the experimenter could opt for either a second try, recalibration, or to move forward with the measurement error regardless. All RMS values were logged. Regardless of drift check implementation, some trials were initiated with large RMS. *Crossings* are quite crude of an outcome measure, but we decided to remove trials that were initiated with a mean measurement error of >5° nonetheless. 44 trials exceeded the threshold, and they were all from healthy controls. For patients with KS, the mean drift check value was 0.9 (range 0.11 – 4.34), and mean *SD* was 0.45 (range 0.03 – 5.18). For controls, the mean drift check value was 0.9 (range 0.09 – 4.97), and mean *SD* was 0.53 (range 0.03 – 6.0).

Outlier removal

Table S3. *Linear mixed-effects coefficient estimate and test-statistics (*t, p*) for outcome measures on the Copy Task predicted by factors Group, Condition, and Group * Condition with and without inclusion of outliers (*N *observations for controls (HC) and patients (KS).*

		**Outliers included**			**Outliers excluded**		
	**N outlier (HC, KS)**	**Estimate**	**t**	***p***	**Estimate**	**t**	***p***
**Outcome variable**							
Success rate	2 (2,0)						
- Group		-0.02	-1.53	.133	-0.03	-2.16	.037*
- Condition		-0.09	-3.37	.002**	-0.07	-2.81	.008**
- Group*Condition		-0.10	-2.77	.009**	-0.12	-3.03	.004**
Speed score	5 (3,2)						
- Group		3.19	5.57	<.001***	2.66	6.05	<.001***
- Condition		1.63	2.97	<.005**	1.20	2.12	.04*
- Group*Condition		3.73	4.65	<.001***	4.21	5.1	<.001***
Number of crossings	0						
- Group		2.17	3.47	.001**			
- Condition		-5.64	-12.1	<.001***			
- Group*Condition		-2.34	-3.44	.001**			
Dwell time per crossing	7 (3,4)						
- Group		136.93	2.92	.004**	104.81	2.25	.025
- Condition		1296.85	4.97	<.001***	1026.71	6.96	<.001
- Group*Condition		45.13	0.12	.91		NA[A]	NA[A]
Number of crossings per correct	2 (0,2)						
- Group		0.71	4.08	<.001***	0.56	3.85	<.001***
- Condition		-0.88	-8.65	<.001***	-0.88	-9.64	<.001***
- Group*Condition		-0.24	-1.59	.12	-0.15	-1.11	.27
Dwell time per correct	6 (3,3)						
- Group		0.71	4.61	<.001***	0.45	4.99	<.001***
- Condition		0.41	2.16	.039*	0.29	1.7	.096
- Group*Condition		0.27	0.97	.34	0.62	2.48	.017*

*$p \leq 05$, **$p \leq .01$, ***$p \leq .001$

[A] After outlier exclusion, the linear mixed-effects model failed to converge, suspectedly because the removal of seven participants led to insufficient data to make predictions. We simplified the model by removing the (earlier non-significant) interaction effect, after which the main effects were again observed.

Discussion

Forward Span analysis

Table S4. *Linear mixed-effects coefficient estimates and raw* p-*values for additionally analysed factors (fixed covariates are level of education and age, and forward span score of interest) within the linear mixed-effects regression models to predict sampling behaviour (crossings and dwell time per correct placement) for the patients with Korsakoff's Syndrome on the Copy Task split on condition (baseline, high cost).*

	**No. of crossings per correct placement**				**Dwell time per correct placement**			
	**Baseline**		**High cost**		**Baseline**		**High cost**	
**Digit Span – FW span**	**Est.**	**Raw p**	**Est.**	**Raw p**	**Est.**	**Raw p**	**Est.**	**Raw p**
N=24								
Education	-0.088	.622	0.075	.280	0.080	.586	0.138	.424
Age	0.038	.184	0.011	.303	0.040	.096.	-0.033	.229
FW Span	-0.102	.622	-0.068	.396	-0.160	.352	-0.076	.703
**Corsi – FW span**								
N=23								
Education	-0.258	.146	0.052	.489	-0.062	.683	0.086	.637
Age	0.017	.568	0.016	.220	0.035	.187	-0.029	.356
FW Span	-0.401	.062	0.005	.953	-0.225	.219	-0.018	.933

Note. Digit Span – FW span = forward span on the WAIS IV Digit Span, Corsi – FW span = forward span on the Corsi Block Tapping Test. *$p \leq .05$.

Ineffective crossings

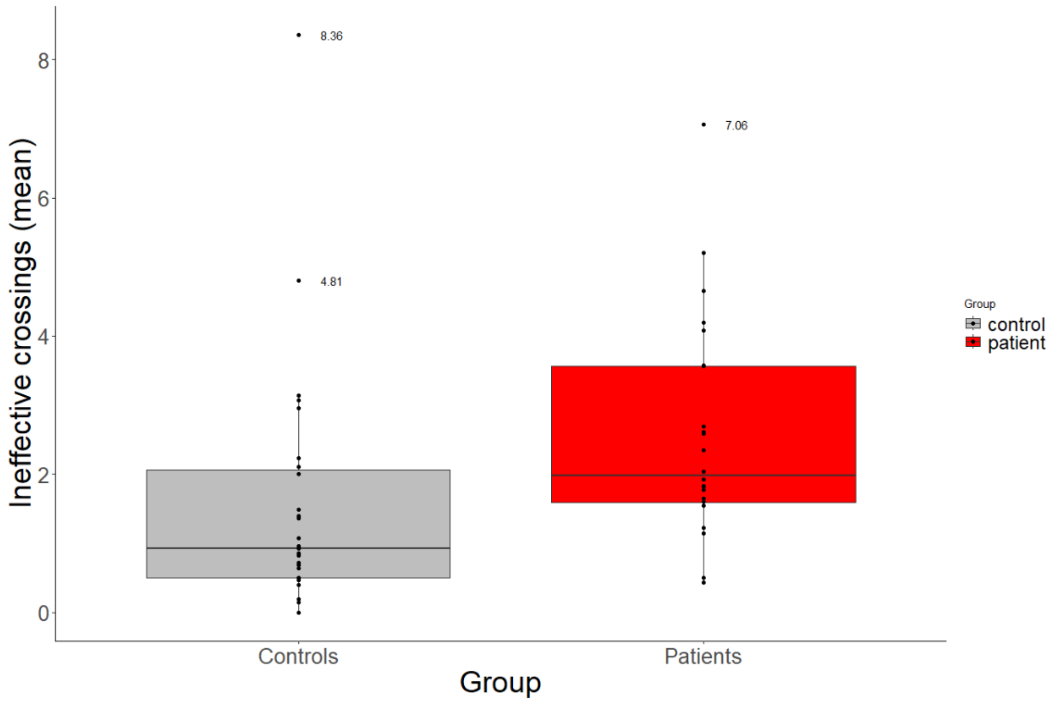

Figure S3. The aggregated number of ineffective crossings in the high cost condition (in baseline, the example was not occluded, and therefore, all crossings could be used for sampling) for controls (grey) and patients with Korsakoff's syndrome (red). The number of ineffective crossings differed significantly across groups (Mann-Whitney-Wilcoxon U=140099, p<.001, rank-biserial correlation r=-0.31). Black dots represent outcomes of individual participants. Outlier values (1.5*interquartile range) are indicated.

References

1. Kessels, R.P.C.; Nys, G.M.S.; Brands, A.M.A.; Berg, E. van den; Zandvoort, V.M.J.E. The Modified Location Learning Test: Norms for the Assessment of Spatial Memory Function in Neuropsychological Patients. *Arch. Clin. Neuropsychol.* **2006**, *21*, 841–846, doi:10.1016/j.acn.2006.06.015.
2. Kessels, R.P.C.; Bucks, R.S.; Willison, J.R.; Byrne, L.M.T. *Location Learning Test (3th Ed.).*; Hogrefe Uitgevers: Amsterdam, 2014;
3. Bouma, A.; Mulder, J.; Lindeboom, J.; Schmand, B.; Brein en Cognitie (Psychologie, FMG) *Handboek neuropsychologische diagnostiek*; Pearson, 2012;
4. Saan, R.J.; Deelman, B.G. De 15-Woordentests A en B. Een voorlopige handleiding (Intern rapport). 1986.
5. Wechsler, D. *WAIS-IV-NL: Wechsler Adult Intelligence Scale – Nederlandstalige Bewerking. Technische Handleiding.*; 4th ed.; Pearson Assessment and Information BV.: Amsterdam, 2012;
6. Corsi, P.M. Human Memory and the Medial Temporal Region of the Brain [PhD Thesis]. *Montr. McGill Univ. Montr.* **1972**, 85.
7. Kessels, R.P.C.; van Zandvoort, M.J.E.; Postma, A.; Kappelle, L.J.; de Haan, E.H.F. The Corsi Block-Tapping Task: Standardization and Normative Data. *Appl. Neuropsychol.* **2000**, *7*, 252–258, doi:10.1207/S15324826AN0704_8.
8. Brunetti, R.; Del Gatto, C.; Delogu, F. ECorsi: Implementation and Testing of the Corsi Block-Tapping Task for Digital Tablets. *Front. Psychol.* **2014**, *5*.
9. Claessen, M.H.G.; van der Ham, I.J.M.; van Zandvoort, M.J.E. Computerization of the Standard Corsi Block-Tapping Task Affects Its Underlying Cognitive Concepts: A Pilot Study. *Appl. Neuropsychol. Adult* **2015**, *22*, 180–188, doi:10.1080/23279095.2014.892488.
